# Georeferenced dataset of maritime piracy in the Gulf of Guinea from 2010 to 2021

**DOI:** 10.1038/s41597-023-02706-x

**Published:** 2023-12-07

**Authors:** Ricardo Moura, Nuno Pessanha Santos, André Rocha, Victor Lobo, Miguel de Castro Neto

**Affiliations:** 1 Centro de Matemática e Aplicações (Nova Math), Universidade Nova de Lisboa, 2829-516 Caparica, Portugal; 2 Portuguese Navy Research Center (CINAV), Portuguese Naval Academy (Escola Naval), Almada, 2810-001 Portugal; 3 Portuguese Military Research Center (CINAMIL), Portuguese Military Academy (Academia Militar), Lisbon, 1169-203 Portugal; 4 NOVA Information Management School (Nova IMS), Universidade Nova de Lisboa, Lisbon, 1070-312 Portugal

**Keywords:** Geography, Developing world

## Abstract

Piracy has been a global concern and a threat to the safety of people performing maritime trade around the globe. Since ancient times maritime piracy has been a common practice that, unfortunately, has not ended in the current days. A georeferenced dataset providing the position, meteorologic conditions, and a description of the occurrence can provide essential information for analyzing this global phenomenon. The dataset focuses on the Gulf of Guinea (GoG) as an area dominated by corruption and weak supervision capacity by the local authorities. The time interval considered in this paper is between 2010 and 2021. Using this simple dataset, it is possible to analyze attributes such as when the piracy occurred or if the illegal activity involved deaths or kidnapping. The accuracy of the data was guaranteed by cross-referencing data sources, so we have 595 pirate attacks accurately described. This dataset can easily be used for data mining, allowing further analysis of the patterns and trends of pirate attacks in the GoG over time.

## Background & Summary

Maritime piracy is a global phenomenon that threatens worldwide shipping and maritime trades. The International Maritime Bureau (IMB), a division of the International Chamber Of Commerce (ICC), reported 195 piracy incidents worldwide in 2020, with a notable focus on the Gulf of Guinea (GoG) area^1^, with 81 incidents^2^. This high number of occurrences is mainly motivated by a weak government system, lack of economic conditions and opportunities, and because it is one of the busiest shipping lanes in the world, with a high volume of oil and other goods being transported through the region^3^. On the other hand, Southeast Asia has experienced a significant decrease in piracy incidents, but everything can change, and we must ensure proper maritime surveillance^4^.

Maritime piracy has a significant economic impact. In every situation where an opportunity exists, someone wants to take it. With most commercial trades made by sea and without proper coastal state water surveillance, opportunities exist, and piracy increases. As a natural consequence for the ships that must operate near those waters, the insurance costs increased, representing approximately 51% of all the operating costs in certain areas^5,6^. Piracy is a persistent and evolving threat, and we must analyze it to decrease and mitigate the existing risk. Knowledge is never enough, and more efforts should be made to acquire more data. The first step to evolving and applying the proper mitigation measures is obtaining knowledge from the existing data.

A good example of well-succeeded operations is the European Union operation ATALANTA near the Somali waters, which has achieved a high level of success and obtained knowledge that can be used to combat piracy near the GoG^7^. Unlike Somali pirates, who mainly focus on ransoms, the GoG pirates are known for being very violent, only on stealing the ship’s cargo^5^. The naval presence in the area was increased to combat this violence. Many other measures were taken, such as using an armed team onboard ships or using specific maritime routes considered safer^6^.

**Fig. 1:**
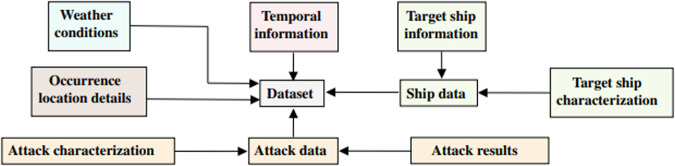
Simplified Illustration of Dataset Content. The dataset^8^ combines information about time, location, weather conditions, attacks, and ships into a single unified data collection.

The dataset^8^ describes 595 reported pirate attacks in GoG between 2010 and 2021. This data was collected from multiple sources, such as the IMB^9^, the International Maritime Organization (IMO)^10^, and the Copernicus Marine Environment Monitoring Service^11^ and then compared and preprocessed to ensure its accuracy. After preprocessing the data, we could construct a database characterizing the georeferenced occurrences near the GoG. The dataset^8^ contains information about time, location, weather conditions, attack data, and ship data. Access to the original data files can be made through the following links: (i) IMB (https://www.icc-ccs.org/index.php/piracy-reporting-centre), (ii) IMO (https://www.imo.org/en/OurWork/Security/Pages/Piracy-Reports-Default.aspx), and (iii) Copernicus (https://data.marine.copernicus.eu/products). The dataset^8^ content will be described in detail in the *Data Records* section. A graphic representation of the dataset^8^ can be seen in Fig. 1.

This information may be valuable for understanding the patterns and trends in maritime piracy attacks on the West African coast. It can aid in developing strategies and policies to prevent future attacks and protect ships in the region. This subject must be studied since any existing opportunity will increase the occurrences near the GoG again. As described, the dataset^8^ was preprocessed to be simple and intuitive, with all the information easily accessible and understandable.

## Methods

The dataset^8^ was created considering an extensive analysis of the IMO and IMB records from 2010 to 2021 (11 years). As happens in any dataset creation, extensive data pre-processing and validation were carried out to ensure the accuracy of the data. Some scripts were created using Python to make this pre-processing easier. All the adopted methods to guarantee the dataset accuracy, including all the data processing, will be described in this section in detail.

### Data cross-checking and data merging

The obtained data were primarily cross-checked and compared to get a more accurate dataset. It is important to note that IMO^12^ and IMB^13^ have a different number of registered attacks, mainly due to the existing agreements between the shipping companies and entities, and that their periodicity and content are different. This makes data cross-checking and data merging more challenging. Despite this, independent from the source, the data followed the same trend^14^. According to their periodicity, these reports are available online in Portable Document Format (PDF).

In the case of the IMO reports, these are created monthly and include the following information:

- Ship name/Ship type/State flag/Gross tonnage/IMO Number (unique ship identifier);
- Date/Time;
- Incident position;
- Incident details;
- Consequences for crew, ship, and cargo;
- Action that the master and the crew took;
- Was the incident reported to the coastal authority? Which one?;
- Reporting state or international organization;
- Coastal State Action Taken.

In the case of the IMB reports, these are created annually and include the following information:

- Ship name/Ship type/State flag/Gross tonnage/IMO Number (unique ship identifier);
- Date/Time;
- Incident position;
- Navigation status;
- If the pirates successfully boarded the ship or it was just an attempt;
- A brief text description of the incident.

**Fig. 2:**
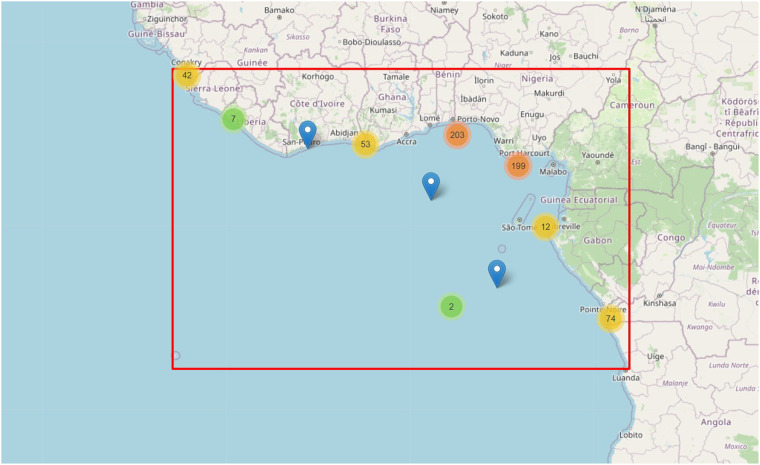
Area of the pirate attacks in the GoG from 2010 to 2021 (red rectangle). The colored circles represent the clustering of the attack by area.

The data were merged and standardized, and any inconsistencies were addressed to ensure the accuracy of the resulting database. For the date and time described on each one of the dataset entries, it is essential to understand the existing meteorological conditions that have allowed the incident or attack. The most significant meteorological conditions for this action at sea are wind speed, wave height, and rainfall level. Datasets from the online Copernicus Ocean Products^15^ were accessed to acquire this information. These datasets provide information services based on satellite observation and *in situ* data. The geographical area is shown in Fig. 2 since all the attacks in the considered time interval have occurred inside the rectangle shown in that map. An initial geographic clustering was performed, and the centroids are represented in Fig. 2 (colored circles), where one can easily see the approximate location of the attacks. An interactive map has been made available for more detailed information on the attacks, including the location of all attacks in the defined period and location. Individual information about the date of the occurrence, classification of the attack, and type of weaponry used are available by clicking on each marker, which displays a pop-up window containing that information. The interactive map is publicly available and can be accessed online at tiiny.host (https://piracygulfguinea.tiiny.site/).

The meteorological data from Copernicus are available in the Network Common Data Form (NetCDF)-4, which can be accessed easily using the NetCDF Python library. This format organizes the scientific data (variables) in a hypercube, which is very useful, *e.g*., to obtain multiple layers of information relating to a specific geographic position and date. Since the available data are discretized in space, and the attack positions do not match entirely with them, the nearest one was selected after calculating the distance to each of the nearby points.

It is important to note that these Copernicus meteorological data are not retrieved from actual measurements (*in situ*) using, *e.g*., buoys but are based on numerical models (Ocean models) that describe the properties of the ocean and all its surrounding factors. These models are fundamental since they can describe the state of the ocean in the past, present, and future^15^. The model accuracy was guaranteed by reanalysis since the Copernicus used model was already corrected by comparing the forecasted and their actual available values^15^.

### Missing data

As described before, some of the considered report variables, such as the distance to the coast, state flag, and criminals’ number, do not match, or this information is unavailable. Thus, we considered all unmatched values as missing data. For each one of the missing data values, it was essential to ensure that we obtained this data with the necessary accuracy.

**Fig. 3:**
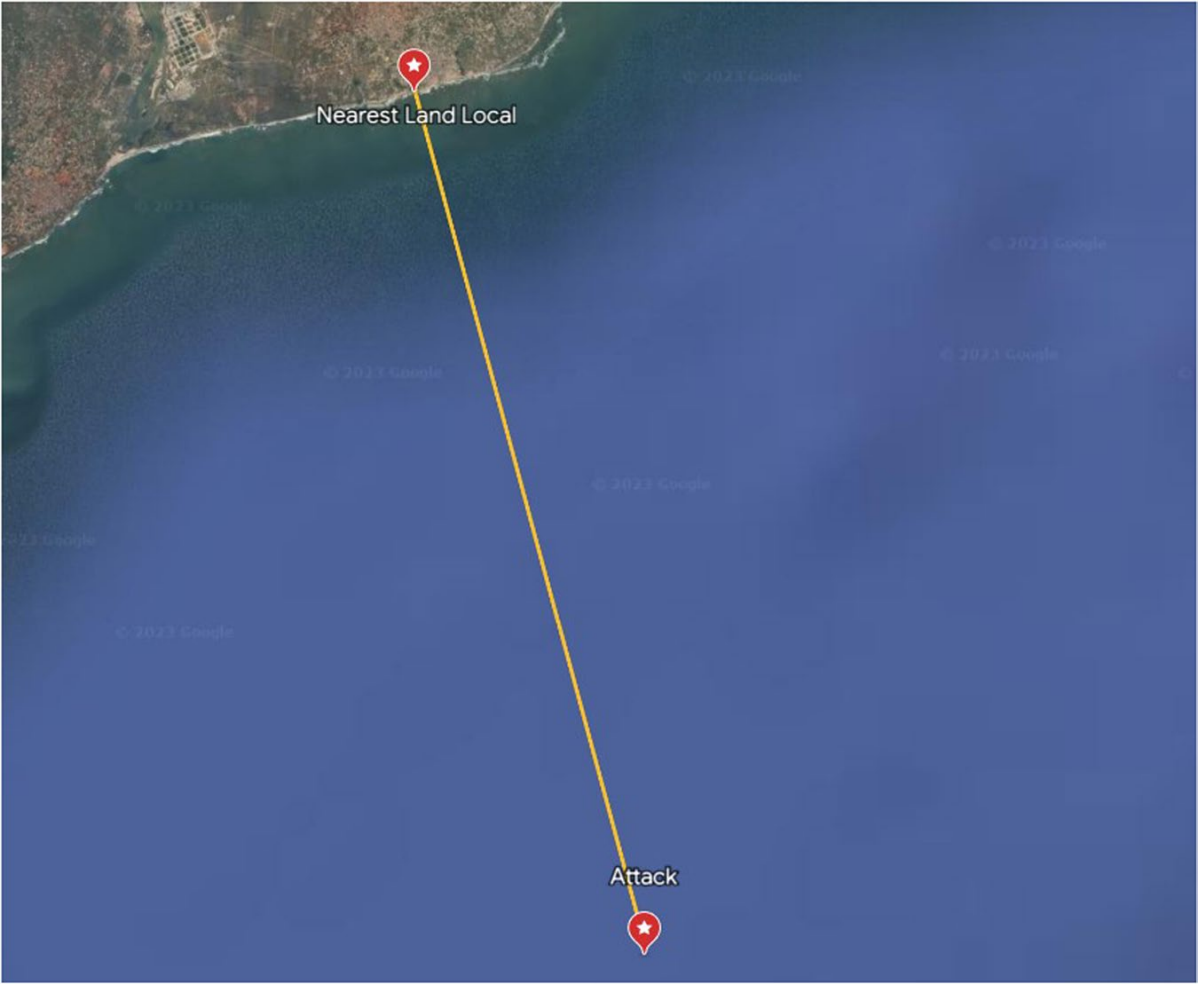
Example of the procedure used to obtain the distance from the coast to a piracy event. All of the distances were acquired using an identical procedure.

The geographic position was considered for all the dataset^8^ entries to obtain the missing distance from the coast. Each point was inserted into the Google Earth Pro^16^, and the nearest point on land coordinates was acquired, as described in Fig. 3. After acquiring the geographic coordinates, the *Haversine* distance was used^17,18^, which gives the shortest distance between two points on the surface of the Earth, taking into account its curvature. This distance is given by:

1 $$d=2r\;\arcsin \left(\sqrt{{\sin }^{2}\left(\frac{{\phi }_{2}-{\phi }_{1}}{2}\right)+\cos ({\phi }_{1})\cos ({\phi }_{2}){\sin }^{2}\left(\frac{{\lambda }_{2}-{\lambda }_{1}}{2}\right)}\right)$$

where *d* is the distance between the two points in kilometers, *r* is the average radius of the Earth (considered 6,371 km), *ϕ*_1_ and *ϕ*_2_ are the latitudes of the two points in radians, and *λ*_1_ and *λ*_2_ are the longitudes of the two points in radians.

To fill in the remaining missing data (state flag and the number of criminals), we utilized a *k*-nearest neighbors classification method^19^. This approach involves using the Euclidean Distance^20^ with all the non-missing variables and using indicator variables for all non-numeric variables. Given that the GoG is located near the equator, where 1 degree of latitude and 1 degree of longitude are both approximately equal to 1 nautical mile, we can treat latitude and longitude as if they were Cartesian coordinates for this case. After testing the algorithm with the non-missing data for the number of neighbors between 1 and 7, it was determined that the best accuracy was obtained when the number of neighbors was set to 3 (*k* = 3). We then used the Euclidean distance to determine the three nearest neighbors and selected the most common value occurrence with the shortest distance to fill in the missing data.

### Data elimination

To ensure the accuracy of the obtained dataset, we had to eliminate entries with inaccurate or missing variables in the analyzed reports. All the dataset entries were eliminated if their respective reports did not have the following data:

- The geographical position, or only having one of its coordinates available;
- The time of the day during which the attack occurred;
- Ship status (sailing, anchored, or docked);
- The name and type of the ship simultaneously.

Since these variables are essential to characterize the attack, and we are focusing on an accurate dataset, we had to discard and eliminate those entries.

### Variable creation, transformation and combination

To obtain an understandable dataset that is simple and intuitive to analyze, we have performed some variables creation, transformation, and combination. All the geographic, meteorological, attack, and ship data were processed, as described in detail.

#### Latitude and longitude

To georeference all the coordinates in the dataset, it was chosen to convert them from degrees and minutes to decimal degrees. The latitude in decimal degrees (*Lat*_*D*_) and the longitude in decimal degrees (*Lon*_*D*_) are given by:

2 $$La{t}_{D}=signa{l}_{Lat}\cdot \left(Degrees+\frac{Minutes}{60}\right)$$

3 $$Lo{n}_{D}=signa{l}_{Lon}\cdot \left(Degrees+\frac{Minutes}{60}\right)$$

where *Degrees* is the integer degree value, *minutes* is the respective minute value, and *signal*_*Lat*_ and *signal*_*Lon*_ are −1, when the coordinates are respectively indicating South and West, or 1, otherwise. Including seconds in the coordinates is unnecessary for the dataset and could introduce unnecessary variability. Therefore, only the degrees and minutes were used to georeference the coordinates in the dataset.

#### Wind categorization

To divide the wind into different scales of intensity (discretize its intervals), the *Beaufort* scale was used^21^. This scale allows direct mapping considering its speed and the effects on sea and land. Since the recorded dataset values only vary between 0.9–8.4 *m/s*, we have only considered the following *Beaufort* scale values:

- **Light air**: 0.5–1.5 *m/s*;
- **Light breeze**: 1.6–3.3 *m/s*;
- **Gentle breeze**: 3.4–5.4 *m/s*;
- **Moderate breeze**: 5.5–7.9 *m/s*;
- **Fresh breeze**: 8.0–10.7 *m/s*.

#### Wave categorization

To divide the wave activity into different scales of intensity (discretize its intervals), the *Douglas* scale^22^ was used. Using this scale, it is also possible to characterize the swell based on the wave height. Since the recorded dataset values only vary between 0.1–2.4 *m*, we have only considered the following *Douglas* scale values:

- **Smooth**: 0.1–0.5 *m*;
- **Slight**: 0.5–1.25 *m*;
- **Moderate**: 1.25–2.5 *m*.

#### Rainfall categorization

The scale proposed by Albert *et al*.^23^ for the Mediterranean region was used for the rainfall characterization. This scale divides the rainfall into the following levels:

- **Light rain**: Up to 4 *mm/day*;
- **Moderate rain**: 4–32 *mm/day*;
- **Heavy rain**: For values above 32 *mm/day*.

#### Ship type aggregation

In the analyzed reports, 26 different types of ships were attacked while crossing the CoG. However, this number was reduced to only four types by aggregating the ships based on their functional characterization and probability of occurrence (we did not consider individually types with a probability ≤2%).

- **Cargo ship**: Ships that are dedicated to transporting large quantities of cargo and containers, including bulk carriers, cargo ships, and supply ships;
- **Oil Ship Transporter**: Ships that transport fuels and oil, including tankers, oil tankers, and product tankers;
- **Chemical Tanker**: Ships that are used for the transport of liquid chemical products;
- **Others**: All those that do not represent more than 2% of the total cases.

#### Risk flag aggregation

The risk flag variable was created to simplify the flag state variable, which presents many possibilities. The risk flag state is divided into three levels of risk based on the number of occurrences recorded by each flag state. In this way, it is possible to access a simpler variable that is easier to understand. The created levels are:

- **Low**: Flag states with less than 20 occurrences (Marshall Islands, Nigeria, and others);
- **Medium**: Flag states with 20–50 occurrences (China and Malta);
- **High**: Flag states with more than 50 occurrences (Liberia, Panama, and Singapore).

#### Attack classification

Since several attacks involve multiple natures (hijacking, kidnapping, or theft), the classification was simplified only to consider the most severe. The severity scale was defined as follows:

$${\bf{Hijack}} > {\bf{Kidnapping}} > {\bf{Theft}} > {\bf{Unsuccessful}}$$

The attack level variable is obtained by dividing the severity scale into three levels. The three considered levels were defined as follows:

- **Level 1**: Indicates that the attack was unsuccessful;
- **Level 2**: Indicates that the attack was successful and the crime committed was theft;
- **Level 3**: It involves the occurrence of a hijack or kidnapping, in which the ship is taken over by the pirates and led to a location for fuel transfer and/or holding the crew members to be able to wait until a ransom is paid.

#### Meteorology creation

A meteorology variable was created to ease using different methods, *e.g*. Bayesian networks^24,25^. This variable summarizes the weather conditions at the time of the attack, using only three values {1, 2, 3}, as described in Table 1.

**Table 1:** Meteorology variable creation - Wind vs. Rain vs. Wave.

	Wind	Light air	Light air	Light air	Light	Light	Light	Gentle	Gentle	Gentle	Moderate	Moderate	Moderate	Fresh	Fresh	Fresh
	Rain	Light	Moderate	Heavy	Light	Moderate	Heavy	Light	Moderate	Heavy	Light	Moderate	Heavy	Light	Moderate	Heavy
**Wave**	**Smooth**	1	1	2	1	1	2	2	2	2	2	2	3	3	3	3
	**Slight**	1	1	2	1	2	2	2	2	2	2	3	3	3	3	3
	**Moderate**	2	2	3	2	2	3	2	2	3	3	3	3	3	3	3

#### Dangerousness creation

Another variable created was the dangerousness, which relates to the type of weapon used and the number of criminals involved in the attack. Again, a severity scale was designed to simplify this analysis. Considering the used weaponry type, the severity scale was defined as follows:

$${\bf{Rocket}}\, \mbox{-} \,{\bf{Propelled}}\;{\bf{Grenade}}({\bf{RPG}}) > {\bf{Firearms}} > {\bf{Unknown}} > {\bf{Knives}}$$

The unknown category was considered in the scale since 46% of the incident reports do not specify the weaponry used. This occurrence was considered more severe than the knife and less severe than carrying a firearm since it is usually used in some method of weaponry, and this assumption will not affect the database accuracy. Also, creating a prediction model to fill almost 50% of the database will not bring good approximation results.

The scale adopted for the number of criminals variable was the same used by IMO in their reports, being described as:

- **Low**: 1–4 criminals;
- **Medium**: 5–10 criminals;
- **High**: More than 10 criminals.

Combining all that was described before, it was possible to combine the two variables into one variable, following the structure described in Table 2.

**Table 2:** Dangerousness - Weaponry vs. Number of criminals.

		Weaponry			
		Knives	Unknown	Firearms	RPG
**Number of Criminals**	**Low**	1	2	2	3
	**Medium**	1	2	3	3
	**High**	2	3	3	3

#### Response action level creation

From the IMO and IMB reports^9,10^, a set of records related to actions taken by the ship as countermeasures to face the threat and prevent an attack were available. These variables were represented in a binary code having the following content:

- **Alarm** - Whether the alarm was triggered or not;
- **Assistance from authorities** - Whether or not authorities intervened;
- **Security team** - Whether or not the security team on board intervened when present;
- **Citadel** - Whether the ship’s crew used the citadel during the attack;
- **Evasive Maneuvers** - Whether or not evasive maneuvers were performed or speed changes were made to avoid being boarded by pirates.

As performed before, a new variable named Response Action Level was created to represent the number of actions the target ship used during the attack. The Response Action Level variable was divided into the following three levels:

- **Low**: None-one response action;
- **Medium**: Two-three response actions;
- **High**: Four-five response actions.

The Response Action Level was automatically considered high when the target ship had a security team onboard and, therefore, intervened since it is the most effective measure a ship can perform to avoid piracy^26,27^.

#### Coastal zone creation

Since we are considering the GoG area, there are in that zone some coastal states that have a low number of piracy occurrences. To deal with that, we have created a variable named coastal zone that divides the considered area into the following four zones:

- **Zone 1**: Guinea-Bissau, Guinea, Sierra Leone, and Liberia;
- **Zone 2**: Ivory Coast, Ghana, Togo, and Benin;
- **Zone 3**: Nigeria, Cameroon, and Equatorial Guinea;
- **Zone 4**: Gabon, Sao Tome and Principe, Congo, Democratic Republic of Congo, and Angola.

## Data Records

The dataset^8^ is available at the Figshare repository (10.6084/m9.figshare.c.6829425.v1) and also at the Mar-IA project webpage (https://mar-ia.pt/). The repository provides access to the following files:

- **unprocessed_piracy_guinea_gulf.xlsx:** Initial dataset in Excel, without any pre-processing;
- **unprocessed_piracy_guinea_gulf.csv:** Initial dataset but using comma-separated values format;
- **piracy_guinea_gulf.xlsx:** The pre-processed dataset in Excel format;
- **piracy_guinea_gulf.csv:** The pre-processed dataset using comma-separated values format;
- **piracy_guinea_gulf.pickle:** The pre-processed dataset using the pickle format. The pickle format is essential as it preserves all properties created during Python processing.

Due to copyright limitations, only IMO attack reports will be made directly available in the repository. The rest of the information can be accessed using the previously provided links or consulting the unprocessed_piracy_guinea_gulf.xlsx or unprocessed_piracy_guinea_gulf.csv files where all the information was merged before the pre-processing.

After all the preprocessing and variable creation, we obtained an accurate dataset^8^ of 595 entries with 38 variables (columns), as described in Table 3. As described in the *Background and Summary* section, the variables can be divided into the following categories (Fig. 1): temporal information, occurrence location details, weather conditions, target ship information, target ship characterization, attack characterization, and attack results.

**Table 3:** Data records description.

Category:	Variable:	Type:	Description:
**Temporal information**	Period	Categorical	*Day* or *Night*
	Date_Time	String or DateTime	YYYY-MM-DD HH:MM:SS
	Date	String	YYYY-MM-DD
	Year	Integer	YYYY
	Month	Integer	MM
	Season	Categorical	*Summer*, *Fall/Autumn*, *Spring* or *Winter*
	African_Season	Categorical	*Cold and Humid* or *Dry and Hot*
**Occurrence location details**	Lat_D	Float	Latitude of the attack
	Lon_D	Float	Longitude of the attack
	Coastal_State	Categorical	*Angola*,, *Togo*
	Coastal_Zone	Categorical	*Zone 1*, *Zone* 2, *Zone* 3 or *Zone 4*
	Distance_from_Coast	Float	Value in nautical miles (*NM*)
	Navigation_Area	Categorical	*Port Area*, *Territorial Waters* or *International Waters*
**Weather conditions**	Wind_Speed	Float	Value in meter per second (*m*/*s*)
	Wind_Speed_cat	Categorical	*Light air*, *Light breeze*, *Gentle breeze*, *Moderate breeze* or *Fresh breeze*
	Wave_Height	Float	Value in meters (*m*)
	Wave_Height_cat	Categorical	*Smooth*, *Slight* or *Moderate*
	Rain	Float	Value in millimeters per day (*mm*/*day*)
	Rain_cat	Categorical	*Light*, *Moderate* or *Heavy*
	Meteorology	Integer	*1*, *2* or *3*
**Target ship information**	Ship_Type	Categorical	*Cargo ship*, *Oil Ship Transporter*, *Chemical Tanker* or *Others*
	Flag	Categorical	*China*, *Liberia*, *Malta*, *Marshall Islands*, *Nigeria*, *Panama*, *Singapore* or *Other*
	Ship_State	Categorical	*Anchored*, *Docked* or *Sailing*
**Target ship characterization**	Flag_Freq	Categorical	*Low Frequency*, *Medium Frequency* or *High Frequency*
	Response_Action_Level	Categorical	*Low*, *Medium* or *High*
**Attack characterization**	N_of_Criminals	Integer	Number of criminals involved in the attack
	N_of_Criminals_cat	Categorical	*Low*, *Medium* or *High*
	Weaponry	Categorical	*Unknown*, *Knives*, *Firearms*, *RPG*
	Attack_Classification	Categorical	*Unsuccessful*, *Theft*, *Kidnapping* or *Hijacking*
	Hijack	Boolean	1↦ Occurred or 0↦ Not Occurred
	Kidnapping	Boolean	1↦ Occurred or 0↦ Not Occurred
	Attack_Level	Integer	*1*, *2* or *3*
	Dangerousness	Integer	*1*, *2* or *3*
	Summary	String	Small occurrence text description
**Attack results**	Injured	Boolean	1↦ People injured or 0↦ No Injured People
	Dead	Boolean	1↦ People Died or 0↦ No Deaths
	Attack_Success	Boolean	1↦ Succeeded or 0↦ Not Succeeded
	Assistance_from_Authorities	Boolean	1↦ Received or 0↦ Not Received

### Temporal information

The temporal information provides us with valuable information regarding the attack’s time. This information is crucial in understanding the context in which the attacks took place. The set of temporal information variables in our dataset^8^ is as follows:

- **Period**: This is a categorical variable representing the time of the day when the attack occurred - *day* (sunrise to sunset) or *night* (sunset to sunrise);
- **Date_Time**: This variable represents the exact date and time of the attack;
- **Date**: This variable represents the exact date of the attack;
- **Year**: This variable is an integer value representing the year when the attack occurred;
- **Month**: This variable is an integer value representing the month when the attack occurred;
- **Season**: This categorical variable represents the season when the attack occurred. It has four unique values: *summer, fall/autumn, spring*, and *winter*;
- **African_Season**: This categorical variable represents the season when the attack occurred, based on African weather patterns. It has only two possible values: *cold and humid* and *dry and hot*. In the GoG region, there are only two seasons, the hot and dry season from November to March and the cold and humid season from April to October.

### Occurrence location details

The location details provide information about where the attacks occurred and the geographic context of the event. These variables include the country with jurisdiction over the maritime space where the attack occurred, the distance from the attack location to the nearest coast, the navigation area, and a categorical variable indicating the coastal zone where the attack occurred. The set of occurrence location details variables in our dataset^8^ is as follows:

- **Lat_D**: A real value representing the latitude of the attack location expressed in degrees with decimals (with North latitudes being positive and South negative);
- **Lon_D**: A real value representing the longitude of the attack location expressed in decimals;
- **Coastal_State**: A categorical value representing the country with jurisdiction over the maritime space where the attack occurred. We have considered the 15 states located in the region;
- **Coastal_Zone**: A categorical variable indicating which one of the four coastal zones defined in the *Methods* section the attack occurred;
- **Distance_from_Coast**: A real value representing the distance in nautical miles from the attack location to the nearest coast;
- **Navigation_Area**: A categorical value representing the geographic area indicating whether the attack occurred in the *Port Area*, in the *Territorial Waters*, which extends up to 12 nautical miles, or in *International Waters*, which extend beyond 12 nautical miles.

### Weather conditions

The weather variables provide information about the weather conditions during the attack. These variables include wind speed, the height of the waves, and rainfall. The set of weather conditions variables in our dataset^8^ is as follows:

- **Wind_Speed**: A real number representing the wind speed value at the attack location in *m/s*;
- **Wind_Speed_cat**: Categorical variable related to the wind speed created as described in the *Methods* section;
- **Wave_Height**: A real number representing the Wave height at the attack location in *m*;
- **Wave_Height_cat**: Categorical variable related to the wave height created as described in the *Methods* section;
- **Rain**: A real number representing the rainfall at the attack location in *mm/day*;
- **Rain_cat**: A categorical variable related to the rainfall created as described in the *Methods* section;
- **Meteorology**: An integer indicating the type of meteorological conditions created as described in the *Methods* section.

### Target ship information

The variables related to the information of the targeted ship provide information about the type of ship attacked, its flag, and its state of origin. The set of target ship information variables in our dataset^8^ is as follows:

- **Ship_Type**: A categorical value related to the type of attacked ship as defined in the *Methods* section;
- **Flag**: A categorical value related to the country of registration of the ship that defined the internal maritime law by which it is governed;
- **Ship_State**: A categorical value related to the ship’s status when attacked, consisting of only three unique possible values - sailing, anchored, or docked.

### Target ship characterization

The target ship characterization variables provide information about the risk and response action level associated with the ship. The set of target ship characterization variables in our dataset^8^ is as follows:

- **Flag_Freq**: A categorical variable indicating the level frequency of the attacks associated with the ship’s Flag, as described in the *Methods* section;
- **Response_Action_Level**: A categorical variable indicating the level of the response action of the ship, as described in the *Methods* section.

### Attack characterization

This section explains the attack’s results and the violence level used. The variables include the number of criminals involved in the attack, the type of weapons used, and the classification of the attack. Boolean variables are also included to indicate whether the ship was hijacked and whether there were any kidnappings during the attack. Additionally, an integer variable demonstrates the dangerousness level of the attack, and a text field briefly describes the occurrence. The set of attack characterization variables in our dataset^8^ is as follows:

- **N_of_Criminals**: An integer representing the number of criminals involved in the attack;
- **N_of_Criminals_cat**: A categorical variable related to the number of criminals involved in the attack, created as described in the *Methods* section;
- **Weaponry**: A categorical variable indicating the type of weapons used in the attack, including firearms, knives, RPGs, or unknown if this information is not reported;
- **Attack_Classification**: A categorical value of the attack classification consists of the following classes: Unsuccessful, Theft, Hijacking, and kidnapping;
- **Hijack**: A Boolean variable that indicates if the ship was hijacked and the pirates could take control of the ship’s maneuvering;
- **Kidnapping**: A Boolean variable indicating if there were any kidnappings during the attack;
- **Attack_Level**: An integer indicating the level of the attack as described in the *Methods* section;
- **Dangerousness**: An integer indicating the level of danger of the attackers as described in the *Methods* section;
- **Summary**: A small free text field describing the attack.

### Attack results

The category attack results have variables that provide information about the attack’s consequences, including the existence of injured or killed people, whether the attack was successful, and whether the attacked ship received assistance from authorities. The set of attack results variables in our dataset^8^ is as follows:

- **Injured**: A Boolean variable indicating if there were people injured during the attack;
- **Dead**: A Boolean variable indicating if people died during the attack;
- **Attack_Success**: A Boolean variable indicating if the attack was successful;
- **Assistance_from_Authorities**: A Boolean variable indicating whether the attacked ship received assistance from the local authorities.

## Technical Validation

The dataset^8^ was compiled from various sources and underwent rigorous processing to ensure accuracy and completeness. The IMO, IMB, and Copernicus datasets are extensively used and guarantee the best accuracy, considering the difficulty of gathering some variables. Even so, a multi-step approach was adopted to validate the dataset’s technical quality. Firstly, the dataset was meticulously cleaned and filtered to remove duplicate or irrelevant entries. Secondly, inconsistencies and missing values were addressed, and missing values were imputed where possible. Thirdly, the dataset was standardized to ensure consistency and ease of analysis.

An Exploratory Data Analysis (EDA) was performed to evaluate the dataset’s^8^ reliability and usability^28^. This involved analyzing the statistical properties of the dataset, identifying patterns, and visualizing relationships between variables. As described in the *Missing Data* and *Data Elimination* sections, to ensure the dataset accuracy, some inaccurate or missing values were removed and replaced with data with the necessary accuracy. At the same time, low-quality entries were removed since they did not possess the considered essential variables to characterize the attack.

In conclusion, this dataset’s^8^ technical validation process involved multiple cleaning, standardization, and review stages. The exploratory data analysis provided strong evidence of the dataset’s reliability, making it a valuable resource for researchers in this field.

## Usage Notes

The provided dataset^8^ offers various variables that can be analyzed using software packages like R, Python, or MATLAB to identify trends or patterns in piracy attacks over time. Researchers are encouraged to share their code, programs, or data-processing workflows if they may help others understand or use the data.

The *Attack Characterization* section provides information on the attack’s results and the level of violence used, including the number of criminals involved, the type of weapons used, and the classification of the attack. There are also Boolean variables indicating if the ship was hijacked or if there were any kidnappings during the attack. An integer variable indicates the level of the attack, and a text field provides a brief description of the attack. Researchers can use these variables to identify trends or patterns in piracy attacks that occur over time.

Similarly, the Attack Results variables provide information about the consequences of the attack, including the number of people injured or killed, whether the attack was successful, and whether the attacked ship received assistance from authorities. These variables can be analyzed to identify trends in the success of piracy attacks or the level of assistance received by the attacked ship.

Moreover, the dataset’s^8^ text description of the attack allows researchers to develop text-mining algorithms that automatically complete some variables related to the attack characterization and results.

## Data Availability

The code used to generate the interactive visualization of the attacks shown in Fig. 2 is provided in the convert.py (https://github.com/ricardomourarpm/Gulf_of_Guinea_Piracy). To run the provided code, it is possible to run it locally using Python in a Jupyter Notebook or even use the Anaconda Distribution, or you can use it directly online using, e.g., the Google Colab (https://colab.research.google.com/). The Anaconda Distribution (https://jupyter.org/install.html) is usually a good choice since it includes Python, the Jupyter Notebook, and other commonly used scientific computing and data science packages.
